# Fabrication of a spherical inclusion phantom for validation of magnetic resonance-based magnetic susceptibility imaging

**DOI:** 10.1371/journal.pone.0220639

**Published:** 2019-08-05

**Authors:** Jun-Ho Kim, Jung-Hyun Kim, So-Hee Lee, Jinhyoung Park, Seung-Kyun Lee

**Affiliations:** 1 Department of Biomedical Engineering, Sungkyunkwan University, Suwon, South Korea; 2 IBS Center for Neuroscience Imaging Research, Suwon, South Korea; New York University School of Medicine, UNITED STATES

## Abstract

Fabrication of a spherical multi-compartment MRI phantom is demonstrated that can be used to validate magnetic resonance (MR)-based susceptibility imaging reconstruction. The phantom consists of a 10 cm diameter gelatin sphere that encloses multiple smaller gelatin spheres doped with different concentrations of paramagnetic contrast agents. Compared to previous multi-compartment phantoms with cylindrical geometry, the phantom provides the following benefits: (1) no compartmental barrier materials are used that can introduce signal voids and spurious phase; (2) compartmental geometry is reproducible; (3) spherical susceptibility boundaries possess a ground-truth analytical phase solution for easy experimental validation; (4) spherical geometry of the overall phantom eliminates background phase due to air-phantom boundary in any scan orientation. The susceptibility of individual compartments can be controlled independently by doping. During fabrication, formalin cross-linking and water-proof surface coating effectively blocked water diffusion between the compartments to preserve the phantom's integrity. The spherical shapes were realized by molding the inner gel compartments in acrylic spherical shells, 3 cm in diameter, and constructing the whole phantom inside a larger acrylic shell. From gradient echo images obtained at 3T, we verified that the phantom produced phase images in agreement with the theoretical prediction. Factors that limit the agreement include: air bubbles trapped at the gel interfaces, imperfect magnet shimming, and the susceptibility of external materials such as the phantom support hardware. The phantom images were used to validate publicly available codes for quantitative susceptibility mapping. We believe that the proposed phantom can provide a useful testbed for validation of MR phase imaging and MR-based magnetic susceptibility reconstruction.

## 1. Introduction

Static magnetic susceptibility is an important magnetic resonance imaging (MRI) biomarker that carries information about iron deposit in tissue as well as myelin density and hemorrhage [1–3]. In MR-based magnetic susceptibility imaging, gradient echo phase images are converted into static magnetic susceptibility maps of the imaged object through dedicated reconstruction algorithms. This process is known as quantitative susceptibility mapping (QSM). While many reconstruction algorithms have been developed and successfully deployed in clinical settings, many still suffer from reconstruction artifacts and slow computation [4]. In order to validate different susceptibility reconstruction algorithms, geometric phantoms have been widely used [5–7]. In QSM, the dipolar inversion process can only determine relative (difference) susceptibilities; therefore, a validation phantom must contain at least two compartments with different susceptibilities. In addition, the following properties are desirable:

- In order to mimic tissue in an organ, compartments should be nested.
- Non-tissue-mimicking materials, such as air bubbles and structural barriers for compartmental separation, can cause phase errors and should be minimized.
- Compartmental shapes with known analytical dipolar field solutions are beneficial for validation of phase measurement, a pre-requisite of susceptibility reconstruction.

Literature survey indicates that thin cylindrical or elongated ellipsoidal shapes embedded in a larger cylindrical container have been frequently used for validation phantoms [5, 7]. In order to minimize the compartmental barrier material, some authors have used thin plastic or latex balloons to contain the inner-compartment materials [7–9]. Other authors [10] have constructed a gel inclusion phantom where the inner materials were cut from thin cylindrical agarose rods and positioned inside a background gel in a semi-random fashion; in this case no structural barrier was used but the orientation of the cut pieces was uncontrolled.

While these previous phantoms have served their purposes in their respective experiments, in our opinion their designs were not conducive to good control of the compartmental geometry, necessary for reproducible research. Furthermore, predominantly cylindrical or elongated geometry chosen for the inner compartments could limit experiments to imaging on primarily transverse planes with respect to the compartments' long axis. This is because phase images in the longitudinal planes are sensitive to air bubbles/pockets that are prone to form at the top of the elongated compartments. Since the susceptibility-induced phase maps are strongly orientation dependent, a phantom that can be imaged in any scan plane is desirable for volumetric susceptibility imaging validation.

With these points in mind, here we demonstrate fabrication of a spherical phantom with spherical inner compartments ("inclusions"), in which a 10 cm-diameter spherical gelatin body encloses smaller spherical gelatin balls at controlled locations without using foreign barrier or support materials. The inner sphere susceptibility can be controlled independently from the enclosing sphere by doping with paramagnetic contrast agents. The spherical shape of the overall phantom served to eliminate bulk background phase caused by the air-phantom boundary, whereas the spherical inner compartments produced 3D phase variation according to a known analytical solution. These beneficial features hold regardless of the phantom's physical orientation in the magnet bore or the scan plane choice. We demonstrate use of our phantom to validate publicly available QSM reconstruction software.

## 2. Theory

The effect of a spherical susceptibility boundary on MRI phase is well established. Here we review the basic theory to motivate the present research.

When a uniform sphere with magnetic susceptibility χ is placed in an applied magnetic field *B*_app_ in vacuum, the sphere develops magnetization $\mu_{0}M=\left(\frac{\chi }{N\chi +1}\right)B_{app}$, where *N* = 1/3 is the demagnetization factor of a sphere [1]. For biological tissue with |χ| on the order of 10^−5^, this equals for all practical purposes *μ*_0_*M* = *χB*_app_. The magnetization generates a macroscopic magnetic field ${\delta B}_{sphere}=\frac{2}{3}\mu_{0}M$ inside the sphere that adds to the applied field *B*_app_. Importantly, in MRI the proton Larmor frequency *ω*_0_ = *γB*_0_ (γ is the gyromagnetic ratio) inside a magnetized medium is determined not by the total macroscopic magnetic field, but by the magnetic field B_0_ that would exist if a small hypothetical sphere called "Lorentz sphere" is carved out of the medium [11]. This is to exclude the molecular self-field from the frequency shift calculation and has been experimentally verified. Since *δB*_sphere_ is independent of the radius of the sphere, carving out a Lorentz sphere precisely takes away $\frac{2}{3}\mu_{0}M$ from the field shift, leaving 0 for the shift induced by a uniformly magnetized sphere:

$${\delta B}_{0}(inside\;spherical\;medium)=\delta B_{sphere}-\delta B_{Lorentz\;sphere}=0.$$ (1)

This means that the gradient echo image phase inside the sphere vanishes: $\phi_{sphere}^{in}=\gamma \cdot TE\cdot \delta B_{0}=0$, where *ϕ* is the image phase minus any radio-frequency (RF) field-related phase, and TE is the echo time.

A uniformly magnetized sphere also changes the static field outside the sphere. The change is given by the magnetic field of a point dipole located at the center of the sphere, with matched total magnetic moment. Only the component of the field in the direction of *B*_app_ measurably changes the Larmor frequency. The phase due to this field is given by

$$\phi_{sphere}^{out}=\gamma \cdot TE\cdot \frac{\mu_{0}m}{4\pi }\cdot \frac{\left(3\;\cos^{2}\theta -1\right)}{r^{3}}.$$ (2)

Here *m* = *M*⋅*V*_*sphere*_ is the magnetic moment of the sphere (assumed to be parallel to *B*_app_ as is the case for induced moments) with volume *V*_sphere_, and *r*, *θ* are the polar coordinates of the measurement point with respect to the center of the sphere, with *B*_app_ defining the *z* axis.

**Fig 1:**
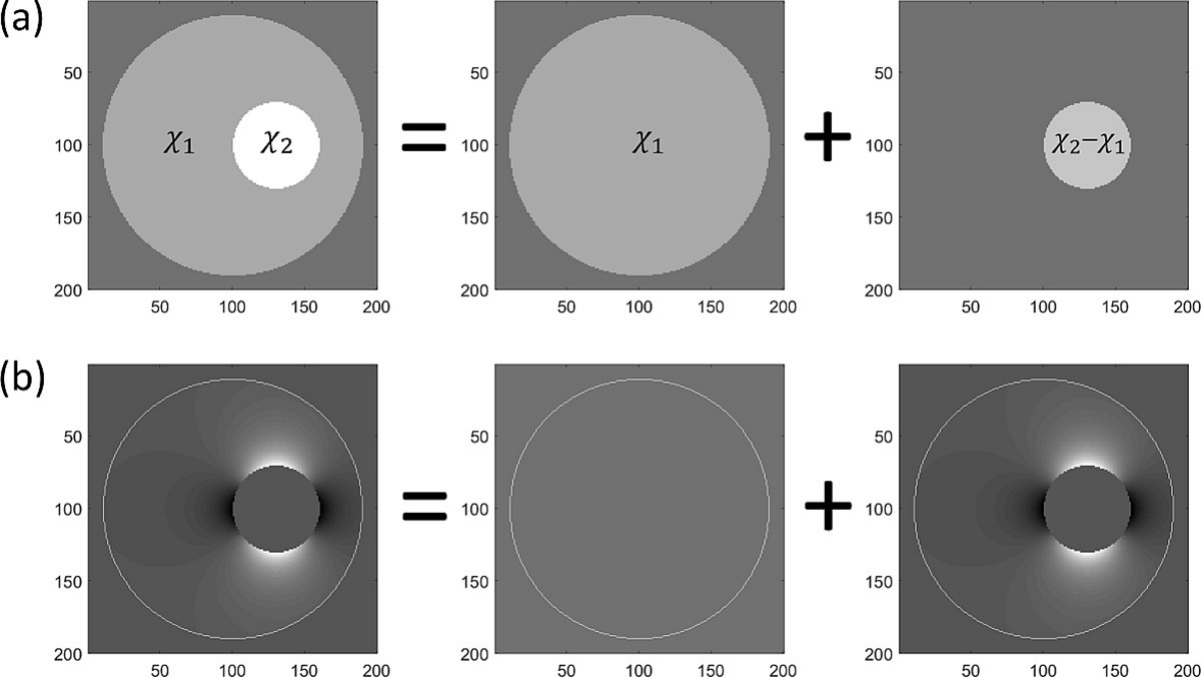
Phase shift in nested spheres. (a) Nested sphere as the superposition of two homogeneous spheres. (b) Corresponding phase shift maps.

A nested sphere where a small homogeneous sphere of susceptibility χ_2_ is embedded in a larger sphere with susceptibility χ_1_ can be thought of as a superposition of the large whole sphere and a small sphere with susceptibility χ_2_-χ_1_. Applying the Lorentz sphere argument to both spheres, we conclude that inside the small sphere, the MRI phase shift is zero. In the space outside the small but inside the large sphere, the phase shift is given by Eq (2) where *m* is the magnetic moment of the small sphere with susceptibility χ_2_-χ_1._ The phase shift for nested spheres is illustrated in Fig 1.

## 3. Materials and methods

### 3.1. Phantom fabrication

**Fig 2:**
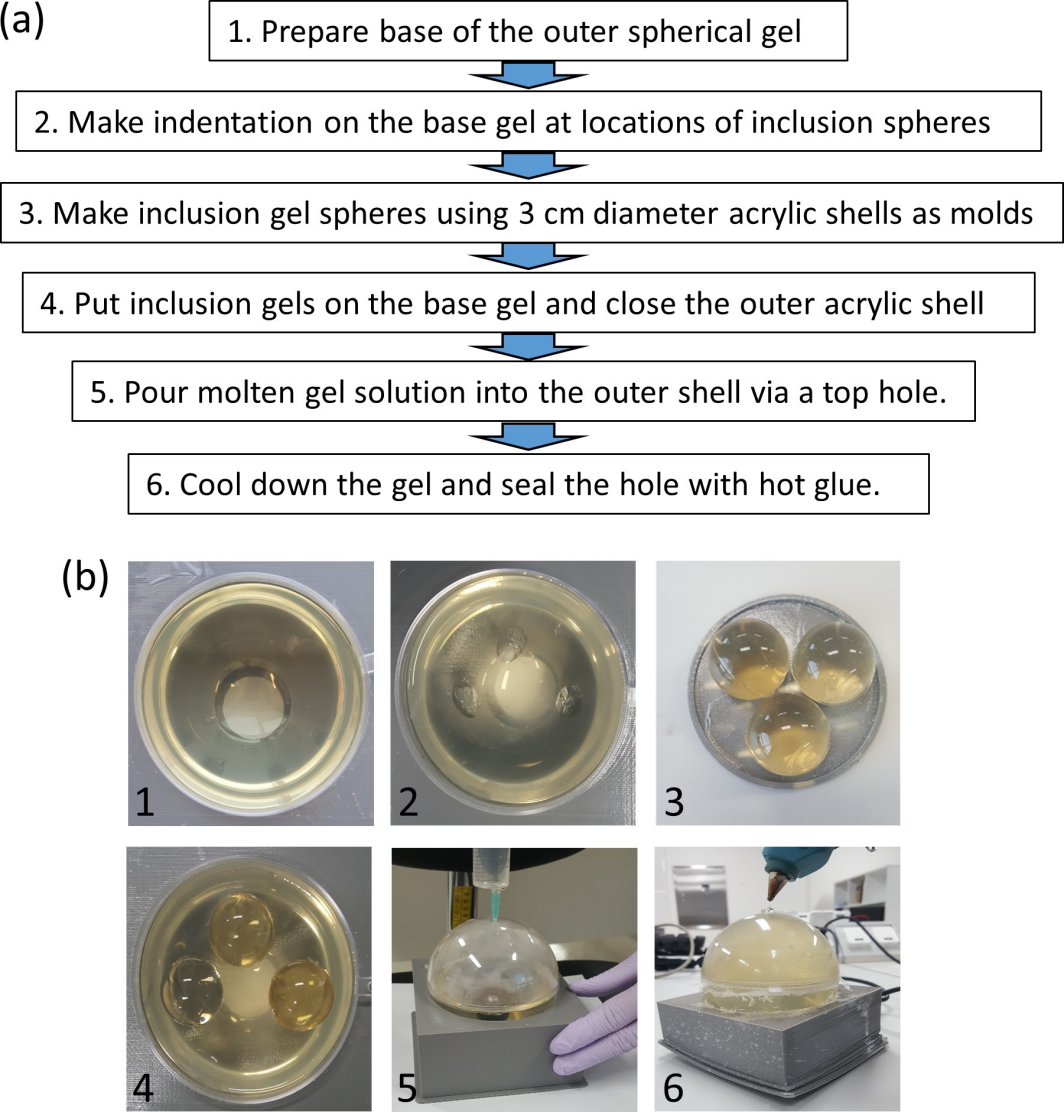
Fabrication workflow. (a) Steps for the fabrication of a spherical inclusion phantom. (b) Pictures of each step. 3D-printed, bowl-shaped holder (grey) had a hole in the middle which is visible in Steps 1, 2, 4.

All the phantom compartments were made out of aqueous solution of gelatin powder (G1890, Sigma Aldrich, St. Louis, Mo, USA). In order to minimize water diffusion between the gel boundaries, the gel solution was mixed with 10% neutral buffered formalin solution which induced crosslinking of the polymers to raise the melting point and reduce the gel porosity [12]. The spherical shape of the whole phantom, 10 cm in diameter, was achieved by building the phantom inside a thin acrylic shell assembled from two hemispheric shells epoxied or pressure-fit together. The spherical inclusions were made by injecting the aqueous solution into a 3 cm-diameter acrylic spherical shell and separating the shell after the solution gelled. Furthermore, a waterproof coating agent (MP131, 3M Company, Maplewood, MN, USA) was applied on the outer surface of the 3 cm inclusions prior to inserting them into the enclosing gel. Detailed steps for phantom fabrication are presented in the following. Fig 2 shows the fabrication workflow, and Table 1 lists the materials used. Among the materials in Table 1, only formalin solution is toxic and requires personal protective equipment for routine laboratory handling.

**Table 1:** Materials used for the fabrication of a 10 cm-diameter gelatin sphere phantom with three inclusions.

Item	Amount used	Model/product description	Manufacturer	Vendor/local distributor URL^a^
Gelatin	75 g	G1890	Sigma-Aldrich, St Louis, MO, USA	https://www.sigmaaldrich.com/
Preservative	15 ml	Germall Plus	Ashland, Covington, KY, USA	www.tonature.co.kr
Formalin solution	8 ml	neutral buffered, 10%	Sigma-Aldrich, St Louis, MO, USA	https://www.sigmaaldrich.com/
Acrylic shell	1 EA (10cm), 3 EA (3 cm)	KUG100, KUG030	Schiller-Plastic, Rechberghausen, Germany	http://www.dnara.kr
Waterproof coating agent	~ 5 ml	MP131 (liquid)	3M Company, Maplewood, MN, USA	https://front.wemakeprice.com/product/179830481?utm_source=google_ss&utm_medium=cpc&utm_campaign=r_sa
Epoxy	~ 2 ml	MagicFix two-part epoxy	PC-Products Co, Allentown, PA, USA	http://item.gmarket.co.kr/Item?goodscode=1139284242
Iron oxide contrast agent	0.12 ml	Molday ION, CL-30Q02-2B (30 mg Fe/ml)	BioPAL, Worcester, MA, USA	http://www.biopal.com/molday-ion.htm
Gd contrast agent	~ 10 ml, as needed	Dotarem (0.5 M)	Guerbet, Villepinte, France	http://www.guerbet.co.kr/fileadmin/user_upload/korea/Contrast_Media/232502_Dotarem_Insert_final_20180321.pdf

a. All URLs were accessed 06/08/2019.

Step 1. Lower layer of the outer sphere

A gelatin solution was prepared by adding 23.4 g of gelatin powder to 300 ml de-ionized water at room temperature and bringing the solution to boil on a hot plate. The solution was then cooled down to 45°C in approximately 60 minutes. At this point the typical amount of solution was 250 ml, to which we added 4.5 ml of liquid Germall Plus (Ashland, Covington, KY, USA) as a non-toxic preservative [12]. In our experience, the preservative-treated gelatin phantom remained mold-free for at least 6 months when kept in a refrigerator. Its magnetic properties over time are discussed in Section 4.4. Shortly after adding the preservative, 2.5 ml of formalin solution (10% neutral buffered, Sigma Aldrich) was mixed with the gelatin solution. The prepared solution, at about 40°C, was poured into a 10 cm diameter acrylic hemispherical shell to a depth of 4 cm, and was cooled down in a refrigerator overnight in an upright position.

Step 2. Defining the inclusion locations

After the 4 cm-deep gel base was congealed, small (1.5 cm diameter, 0.5 cm deep) indentations were made by scooping out the gel with a spoon at locations where the inclusion spheres will be embedded. The shape of the indentations was not precisely controlled; any physical gap between the base gel and the inner spheres due to curvature mismatch can eventually be filled by the gel solution poured at a later step. Our process was effective to embed up to four 3 cm-diameter inclusion spheres on one horizontal plane. Below we describe the process for making a phantom with 3 inclusion spheres.

Step 3. Inner spheres

Three small gelatin spheres were separately made by preparing molten gelatin solution, treated with the preservative and mixed with formalin, as described in Step 1. To control the magnetic susceptibility of each sphere, varying concentrations of paramagnetic contrast agents were added to the solution before cooling down in a refrigerator. In our experiment we have used iron oxide nanoparticles (Molday ION, Biopal, Worcester, MA, USA) at the iron molar density of 0.043 to 0.129 mM, or Gd chelation agents (Dotarem, Guerbet, Villepinte, France) at 2.4 to 7.2 mM. Below we will only describe experiments with the iron oxide particles. The doped gelatin solution was injected by a syringe into a 3 cm-diameter acrylic spherical shell through a hole (~ 1 mm diameter) at the top, at about 38°C. Each sphere was then immersed in a 50 ml beaker filled with the same gelatin solution to prevent air bubble formation at the top of the sphere. The entire beaker was cooled down in a refrigerator for at least 24 hours; a longer congealing time than for the base gel was needed to ensure sufficient solidification of the gel inside the spherical shell before breaking the mold. The two hemispheres of the shell, initially pressure-fitted, could be easily separated, and gelatin spheres could be taken out. The prepared naked gelatin spheres were hand-coated on the surface with a waterproofing liquid (MP131, 3M, Maplewood, MN, USA), and dried for 5 min at room temperature. Before transferring the spheres on to the base gel, the indentations for the placement of the spheres were "flooded" with a shallow layer of the original gelatin solution (~38°C). The small spheres were then placed on the indentations, minimizing air bubbles at the bottom. Note that contact with warm gelatin solution did not melt the congealed gel compartments because of the formalin cross-linking.

Step 4. Upper shell of the outer sphere

At this point the lower part of the outer gel is contained in the lower hemispherical acrylic shell, supporting three inclusion spheres with different magnetic susceptibilities. Next we fitted the upper hemispherical shell onto the lower shell to complete the spherical enclosure. The upper shell, empty at this point, had a drilled hole (~ 1 mm diameter) at the top for gelatin solution injection in the space between the shell and the three small spheres protruding up from the base gel. In most cases, pressure-fitting the two shells at the equatorial joint was sufficient to create leak-proof enclosure. However, certain batches of the acrylic shells required epoxying at the joint (presumably due to manufacturing variabilities) to prevent leaking of the solution in the next step.

Step 5. Gelatin solution fill-in

To fill in the spherical enclosure, a molten gelatin solution with the same composition as the gel base in the lower hemisphere, was syringe-injected into the sphere through the top hole, at the temperature of ~38°C. As in Step 3, formalin fixing ensured that the molten solution did not damage the integrity of the gel base and the inclusions while being poured in the enclosure.

Step 6. Sealing the top

**Fig 3:**
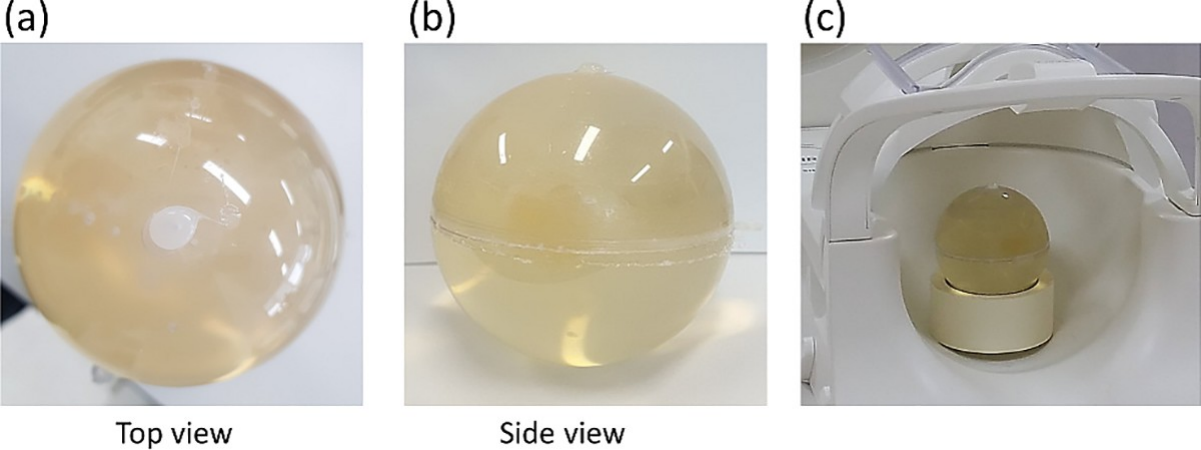
Pictures of the phantom. (a,b) Pictures of the completed spherical phantom. The white blob at the top of the phantom is settled hot glue. (c) Phantom positioned for imaging in a head-neck array coil.

After the whole gel phantom cooled down in a refrigerator for ~12 hours, any void at the top of the sphere was filled as much as possible by adding more gelatin solution and cooling down. Finally, the top hole was plugged with a hot glue. Fig 3 shows the completed phantom.

### 3.2. MRI scan

All MRI scans were performed in a clinical 3T scanner (Magnetom Prisma, Siemens, Erlangen, Germany) with a standard 20 channel head-neck coil. The phantom was placed inside the coil on a roll of masking tape used as a stand. The scanner had a full 2nd order shim coil set which was used to shim the static field before each scan. The actual main magnetic field strength was B_app_ = 2.895 T. Three dimensional multi-echo gradient echo images were obtained with the following scan parameters: Repetition time (TR) = 47 ms, echo times (TE) = 7, 12, 17, 22, 27, 32, 37, 42 ms, flip angle = 20°, bandwidth = 240 Hz/pixel, voxel size = 0.66 × 0.66 × 0.8 mm^3^, matrix size = 176 × 256 × 144, in-plane field of view = 116 × 170 mm^2^, scan time = 12 min 24 sec. Images from different coil channels were complex-combined off-line after subtracting each coil's offset phase determined by extrapolating the multi-echo phases to zero echo time.

In addition, the longitudinal relaxation rate (R_1_), transverse relaxation rate (R_2_), and effective transverse relaxation rate (R_2_*) of the gelatin inclusions were determined by the scanner's standard sequences (Table 2). Specifically, R_1_ was obtained by scanning the phantom multiple times at different repetition times up to 1024 ms with a 90° flip angle in a spoiled gradient echo sequence. R_2_* was obtained by a series of gradient echo scans with a range of echo times (TE = 7 to 60 ms) and a long TR = 2000 ms (approximately 5 times the longitudinal relaxation time of gelatin). R_2_ was obtained similarly as R_2_* but with a spin echo sequence with TE = 15 to 240 ms. The relaxation rate scans were obtained on a single coronal plane with pixel size = 0.78 × 0.78 mm^2^, matrix size = 128 × 128, field of view = 100 × 100 mm^2^. The relaxation rates were obtained by exponential fitting of the magnitude data as a function of TR or TE on a circular ROI of diameter 25 mm covering each inclusion.

**Table 2:** Scan parameters for volumetric phase imaging and single-plane relaxation parameter mapping.

Parameters	Multi-echo GRE (for phase imaging, QSM)	Spoiled GRE (for R_1_)	Spoiled GRE (for R_2_*)	Spin echo (for R_2_)
TR	47 ms	16,32,64,128,256, 512,1024 ms	2000 ms	2000 ms
TE	7,12,17,22,27,32,37, 42 ms	10 ms	7,12,17,22,27,32,37, 42,47,52,57,60 ms	15,30,60,120,240 ms
flip angle	20 deg	90 deg	50 deg	90 deg
slice thickness	0.8 mm	1.5 mm	2.0 mm	2.0 mm
voxel size	0.66 x 0.66 x 0.8 mm^3^	0.78 x 0.78 x 1.5 mm^3^	0.78 x 0.78 x 2.0 mm^3^	0.78 x 0.78 x 2.0 mm^3^
matrix	176 x 256 x 144	128 x 128	128 x 128	128 x 128
FOV in plane	116 x 170 mm^2^	100 x 100 mm^2^	100 x 100 mm^2^	100 x 100 mm^2^
acquisition type	3D	2D	2D	2D
bandwidth	240 Hz/px	260 Hz/px	260 Hz/px	130 Hz/px
acceleration	2	1	1	1
scan time	12 min 24 sec	2 sec ~ 129 sec	4 min 14 sec (per TE)	4 min 14 sec (per TE)
scan plane	transverse	coronal	coronal	coronal

### 3.3. Quantitative susceptibility mapping

Coil-combined multi-echo phase and magnitude images were processed through two publicly available, Matlab (Mathworks, Natick, MA, USA) code packages STISuite v3.0.2 (https://people.eecs.berkeley.edu/~chunlei.liu/software.html, to be called "algorithm 1") and MEDI (http://pre.weill.cornell.edu/mri/pages/qsm.html, updated 03/27/2019, to be called "algorithm 2") for quantitative susceptibility mapping. In algorithm 1, the phase images were first unwrapped by the Laplacian method, and the background phase was removed by spherical mean value-based method with variable radii (V-SHARP)[13] using radius parameter of 35 mm. The resulting three-dimensional local phase map was converted to a susceptibility map by the STAR-QSM method [14] as implemented in the package. In algorithm 2, the image phase was unwrapped by region growing, from which the background phase was removed by projection on dipolar fields [8]. Susceptibility maps were calculated by morphology-enabled dipolar inversion with regularization to suppress susceptibility gradient in regions with low image intensity gradient. The default regularization parameter was used (lambda = 1000), after verifying that changing lambda within a factor of two produced similar results (S6 File).

## 4. Results

### 4.1. Phantom images

**Fig 4:**
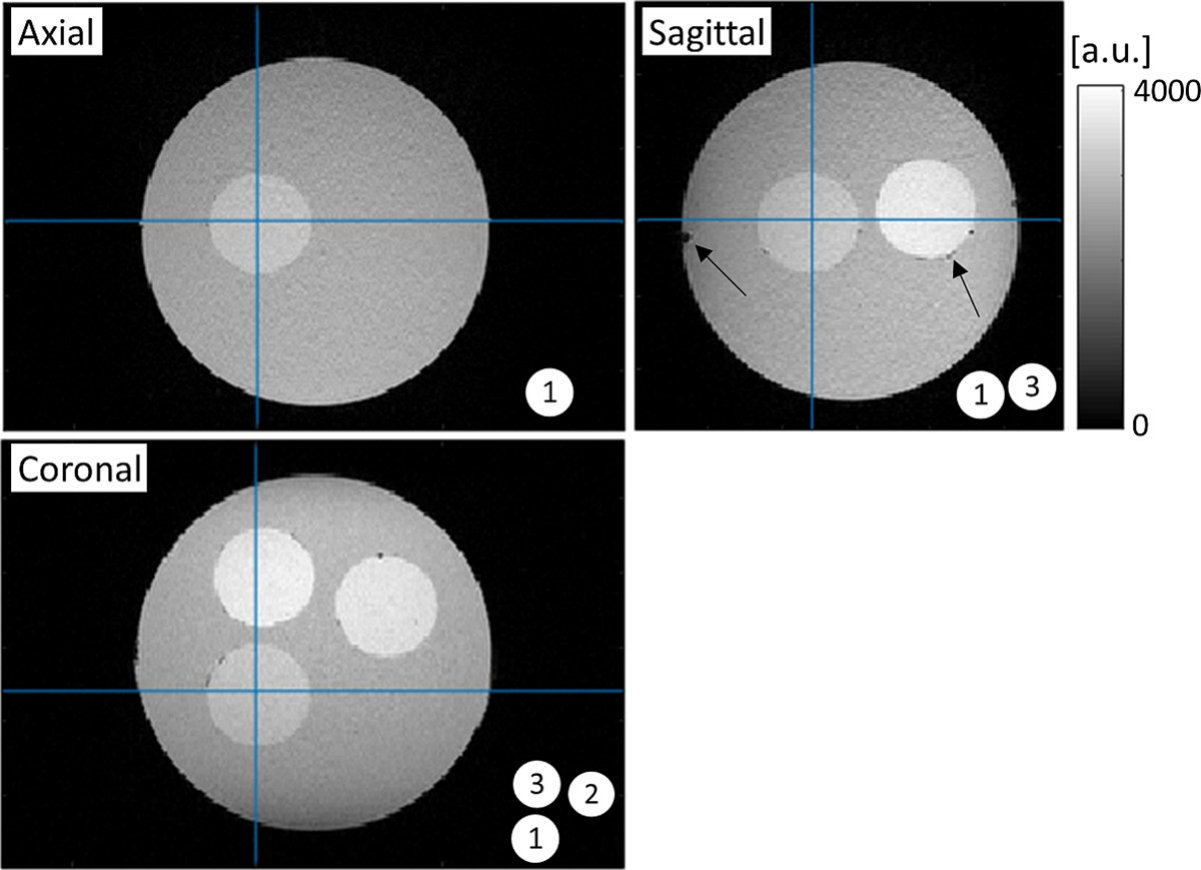
3-plane gradient echo magnitude images of the spherical inclusion phantom. Small air bubbles are visible on the gel boundaries (black arrows). Three inclusions with iron concentrations of 0.043, 0.086, 0.129 mM are indicated as 1, 2, 3, respectively.

Fig 4 shows three-plane gradient echo magnitude images (TE = 7 ms) of the fabricated phantom. The three inclusions with iron molar densities of 0.043, 0.086, and 0.129 mM (marked as 1,2,3, respectively) are visible with sharp compartmental boundaries in the background of the un-doped gelatin. This shows that water diffusion across the boundaries was suppressed effectively. On the other hand, signal voids that are most likely caused by air bubbles are visible on the surfaces of the spheres, as indicated by black arrows.

**Fig 5:**
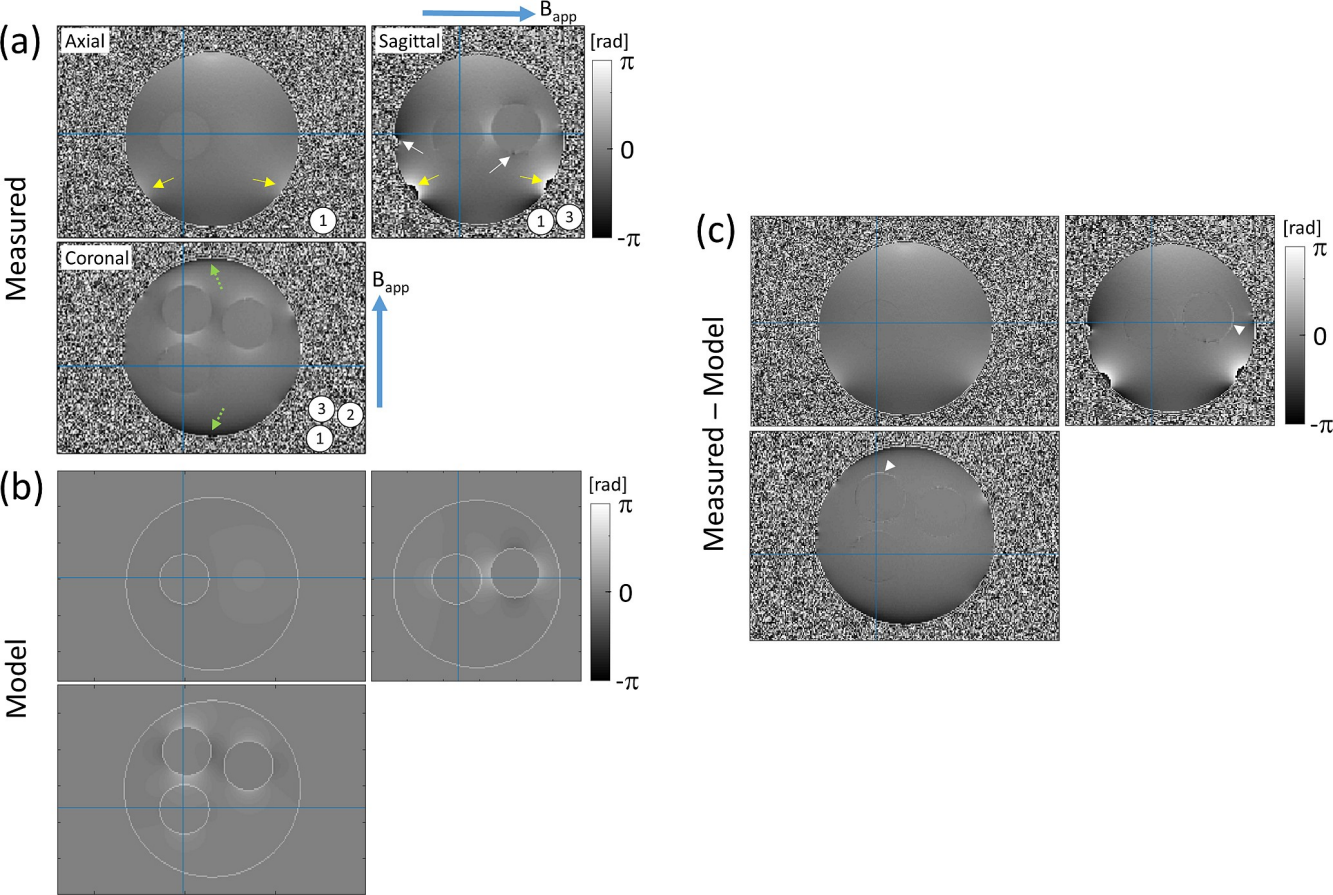
Measured vs model phase maps. (a) Measured phase. Phase variations due to air bubbles (white arrows), phantom stand (yellow arrows), and imperfect shimming (green dotted arrows) are visible. Spherical inclusions (1**–**3) produce dipolar field patterns consistent with the main field (B_app_) direction (blue arrows). (b) Dipolar model phase for inclusion susceptibilities 0.1, 0.2, 0.3 ppm. (c) Phase difference, showing good agreement between model and measurement. White arrowheads indicate error due to model boundary mismatch.

Gradient echo phase images at the same TE are shown in Fig 5. The figure compares the measured phase with the theoretical one based on Eq (2) with susceptibility values of 0.1, 0.2, 0.3 ppm for inclusions 1 to 3. The following observations can be made from Fig 5. First, as predicted the measured phase is close to zero within the inclusion spheres. The mean and standard deviation of the phase for inclusions 1 to 3, calculated within a 25 mm diameter volumetric ROI, were −0.19 ± 0.13 rad, 0.011 ± 0.044 rad, and −0.075 ± 0.061 rad, respectively. Second, the phase around the inclusion spheres displays the characteristic dipolar field patterns with relatively large positive values along the main magnetic field (B_app_) direction, and negative values in the perpendicular directions. The intensity of the phase variation increased from inclusion 1 to 3, in the order of increasing iron concentration and in good agreement with theoretically modelled phase. Finally, the measured phase map in Fig 5 was contaminated by a few unwanted sources: air bubbles (white arrows), contact with the phantom stand (yellow arrows), and slowly-varying background magnetic field left uncompensated by shimming (green dotted arrows). The latter effect could be verified by a separate experiment where the scanner's shim setting was manually changed and corresponding phase changes were observed (S1 Fig).

### 4.2. Relaxation rates

Table 3 shows the measured relaxation rates of the spherical inclusions with different iron oxide concentrations (for details see S2 File). The rates increase approximately linearly with the concentration. Deviation from linearity is likely attributed to errors in controlling the small volumes of the iron oxide solutions. The relaxivities r_1_, r_2_, r_2_* of the contrast agent were determined from the linear regression analysis (S3 File). It is found that the particles act as a strong transverse relaxation agent, while affecting longitudinal relaxation relatively weakly.

**Table 3:** Relaxation rates and relaxivities of the three inclusion spheres.

Inclusion	1	2	3
Iron oxide solution [ml per 250 ml gel]	0.02	0.04	0.06
Iron molarity [μM]	42.9	85.8	128.7
R_1_ [s^-1^ ]	2.24	2.51	2.76
R_2_ [s^-1^]	9.97	17.1	22.2
R_2_* [s^-1^]	10.1	17.3	22.1
r_1_ [s^-1^mM^-1^]	6.06 ± 0.20^a^		
r_2_ [s^-1^mM^-1^]	142 ± 14 ^a^		
r_2_* [s^-1^mM^-1^]	139 ± 17 ^a^		

a. Standard error.

In the literature, r_1_ and r_2_ of the iron oxide particles (in particular, Molday ION) have been reported at different field strengths. For example, the vendor's website (http://www.biopal.com/pdf-downloads/data-sheets/data-sheet-CL-30Q02-2.pdf, accessed 2019/05/24) provides r_1_ = 36 [s^-1^mM^-1^] and r_2_ = 71 [s^-1^mM^-1^] at 0.47 T. Brewer et al [15] reported r_2_ = 178 [s^-1^mM^-1^] at 7T for particles with comparable magnetic core with different functionalization. Given that r_1_ decreases and r_2_ increases with the main magnetic fields [16], our results of r_1_ = 6.1 [s^-1^mM^-1^] and r_2_ = 142 [s^-1^mM^-1^] at 3T are compatible with the literature values.

A significant observation is that for all the inclusions, the transverse relaxation rates and the effective transverse relaxation rates are very close. This reflects homogeneous B_0_ field inside the inclusions, to suppress inhomogeneous spin dephasing, which is a direct consequence of the spherical phantom geometry.

### 4.3. Susceptibility maps

**Fig 6:**
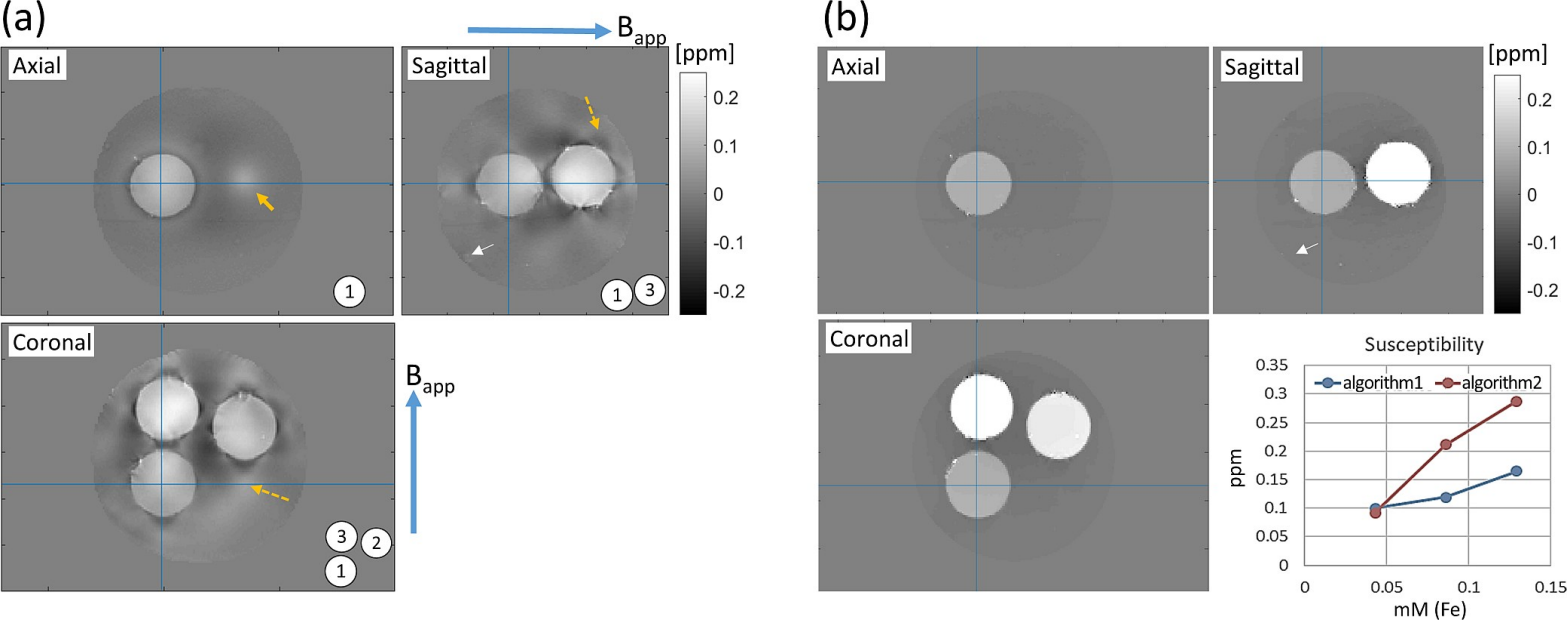
**3-plane quantitative susceptibility maps of the phantom processed with algorithm 1 (a) and 2 (b)**. In (a), streaking artifacts are apparent on the coronal and sagittal planes (orange dashed arrows), giving rise to artificial susceptibility elevation on the axial plane (orange arrow). In both (a) and (b), phase due to the phantom stand produced little susceptibility artifact (white arrows on sagittal planes). The inset of (b) shows the mean susceptibilities of the three inclusions for each processing method. Numerical values and linear fit results are listed in S6 File.

Fig 6 shows the reconstructed susceptibility maps on the same 3 planes as in Figs 4 and 5. With algorithm 1, inclusions 1 to 3 exhibited susceptibility values of 0.100, 0.119 and 0.165 ppm, respectively, averaged over a spherical ROI of diameter 26 mm and referenced to the background gel. With algorithm 2, the corresponding values were 0.092, 0.211, and 0.288 ppm, revealing significant difference between the algorithms. Linear regression (S6 File) of these values as a function of the iron molar concentration yielded the normalized agent susceptibility of 0.758 ppm/mM(Fe) for algorithm 1 and 2.273 ppm/mM(Fe) for algorithm 2. Theoretical susceptibility of the iron oxide particles is different from susceptibility of isolated Fe ions, due to strong spin-spin coupling leading to superparamagnetism. At 3T, the particle's magnetic moment nearly saturates and becomes weakly (weaker than the Curie's law) temperature dependent [17]. A rough estimate can be made from the saturation magnetization of iron oxide nanoparticles of comparable size originally reported (at 310 K) in [18]. Here, the saturation magnetic moment of about 70 emu/g(Fe) can be converted into magnetization *M*_*s*_ = 3.92 A/m/mM(Fe), which translates into an effective susceptibility of *χ* = *μ*_0_*M*_*s*_/*B*_*app*_ = 1.70 ppm/mM(Fe). This is closer to the result of algorithm 2 than algorithm 1.

A more direct evidence that algorithm 1 underestimated the true magnetization of the inclusions can be found from the phase map. Similar to Fig 5, we computed the theoretical dipolar phase map assuming the susceptibility values (0.100, 0.119, 0.165 ppm) obtained from algorithm 1, and subtracted it from the measured phase. The result (S2 Fig) clearly showed residual phase around inclusions 2 and 3 indicating underestimation of susceptibility. While the exact cause of this discrepancy is unclear, we note that paramagnetic susceptibility underestimation in QSM has been reported before [9].

Fig 6 also shows significant qualitative difference in susceptibility maps between the two algorithms. Specifically, algorithm 1 showed streaking artifacts on the coronal and sagittal planes (orange dashed arrows), introducing spurious localized susceptibility elevation on the axial plane (orange arrow). Such artifacts were largely absent in algorithm 2, thanks presumably to morphology based regularization suppressing susceptibility variation within homogeneous regions [6]. Note that subtle brightening in the similar location in the axial-plane phase maps (Fig 5A and 5B) reflects the actual, susceptibility-induced B_0_ variation, and is not related to the streaking artifact. Lastly, we found in both algorithms that the significant phase near the phantom stand shown in Fig 5 (sagittal plane) did not carry over to the susceptibility map as an artifact in Fig 6. This indicates that the background field removal in both QSM algorithms successfully suppressed signals from susceptibility sources outside the phantom.

### 4.4. Stability

**Fig 7:**
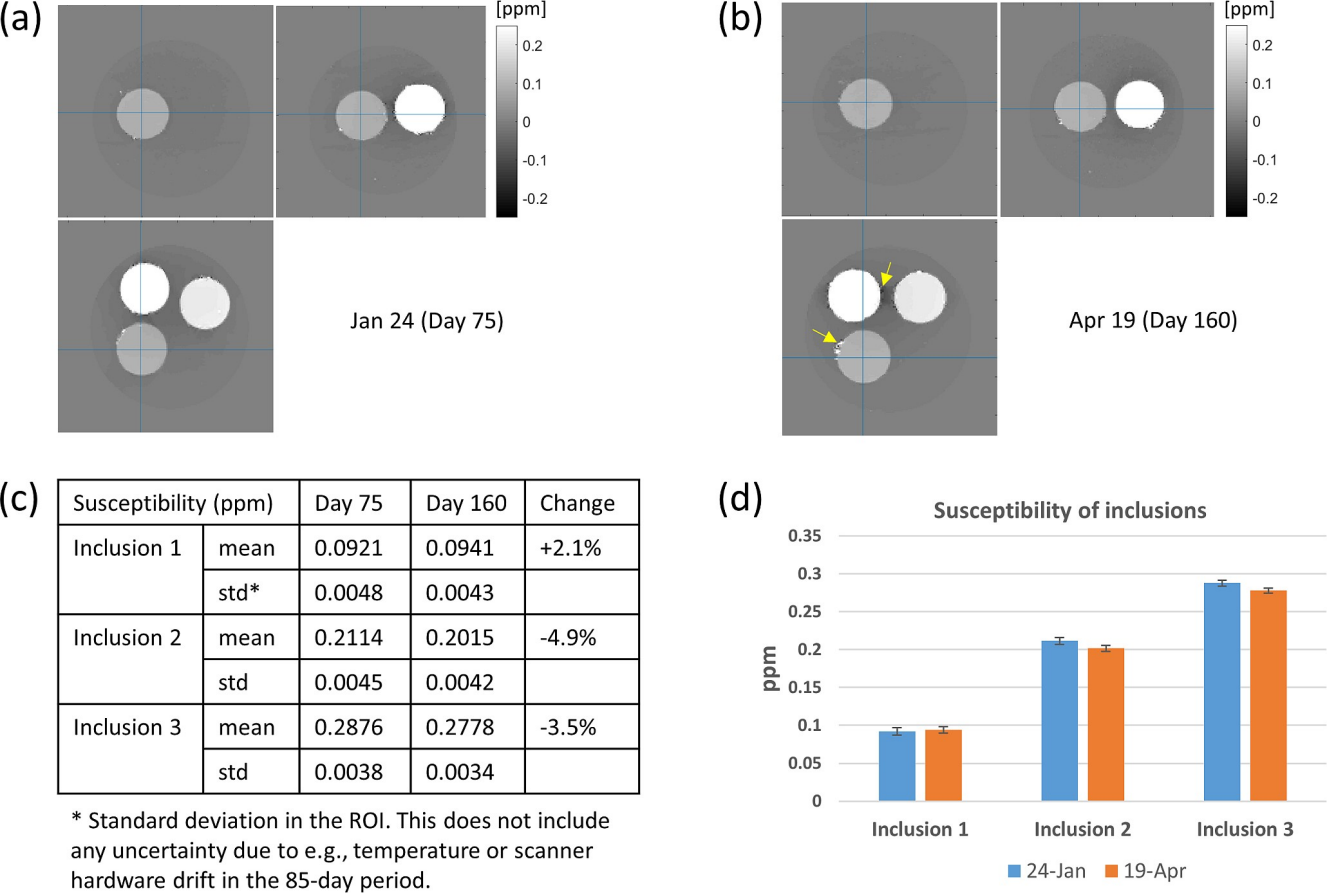
QSM by MEDI obtained with the same phantom 85 days apart. (a-b) 3 plane susceptibility maps; (a) is the same as in Fig 6B. Yellow arrows in (b) indicate additional surface defects. (c) Mean and standard deviation of susceptibility in each inclusion. (d) Plot of susceptibility, with error bars according to 1 std from (c).

In order to assess the stability of the phantom over time, we have conducted QSM and relaxation time measurements on the same phantom 85 days (QSM) and 64 days (relaxation) after the initial experiments (S4 File). Fig 7 shows that the inclusions' mean susceptibility underwent minor change over the 85-day period, namely +2.1%, −4.9%, and −3.5% for inclusions 1**–**3, respectively. This trend was in agreement with R_2_* changes over 64 days (S5 File), which were +0.7%, −2.0%, and −1.3% for the same inclusions. With limited temporal data points, and in the absence of independent scanner drift information, the origin of these changes is unclear. We suspect that water diffusion across compartmental boundaries could be a contributing factor. At present, the proposed phantom appears well-suited for cross-sectional comparative studies, but longitudinal, absolute susceptibility studies may require more work to improve the phantom's stability.

Despite measured susceptibility drifts, the general appearance of the susceptibility maps indicated no evidence of iron settlement at the bottom, or blurring at the spherical boundaries. We noticed, however, that more surface defects formed at the inclusion boundaries in the later-day data (yellow arrows in Fig 7B). We suspect that repeated cooling (for storage) and warming (during scan) could have stressed and possibly cracked the compartmental boundaries. In the future, we plan to investigate storing the phantom at room temperature to prevent thermal expansion-related problems. For this, more conventional (albeit toxic) preservatives such as sodium azide could be more effective than what was used here.

## 5. Discussion

We have demonstrated fabrication of a spherical gelatin phantom with spherical inclusions with different magnetic susceptibilities for possible use as a testbed for MR-based susceptibility imaging. The outer sphere theoretically eliminates background B_0_ inhomogeneity caused by the air-phantom susceptibility boundary, whereas the inner spheres produce well-known volumetric dipolar field patterns for easy verification. Compared to previous susceptibility inclusion phantoms (mostly cylindrical or elliptical, and often utilizing elastic bags), our construction allows more repeatable fabrication. Although non-spherical phantoms have been successfully used for multi-center QSM validation [7], we believe that spherical phantoms have merit for volumetric measurements and phase imaging with available "ground truth". It is our expectation that the proposed spherical inclusion phantom could be of use to validate phase-based imaging and parameter mapping in MRI, including QSM, which has often been done with numerical models or phantoms with less controllable shapes.

For a demonstration, we applied publicly available QSM reconstruction software to calculating susceptibility maps of the fabricated phantom having 3 inner spherical compartments. The results indicated that while the background phase due to the phantom stand was successfully taken care of, the dipolar field inversion process can suffer from streaking artifacts and susceptibility underestimation. While these artifacts have been reported before [9, 19], our phantom provides a way to experimentally explore them in terms of their dependence on image acquisition parameters, such as voxel size, voxel aspect ratio, and scan plane orientation, under a real scanning condition and environment. We emphasize that the purpose of our demonstration was not to address the capabilities of the current QSM algorithms, but rather to present an example of how our phantom can be used to examine such capabilities in future experimental studies. While we have used iron oxide concentrations with relatively easily detectable susceptibility (≥ 0.1 ppm), in the future lower-dose phantoms could be made for more refined tests of QSM.

In our phantom, fabrication errors leading to deviations from the compartmental sphericity came from three main sources: air bubbles, imperfect spherical shape of the acrylic formers, and the seams at the "equators" where two hemispherical shells met. Among these the air bubbles are the least predictable and troublesome in terms of localized phase errors. We have tried to avoid air bubble formation by heating the aqueous gelatin solution before gelation. Also, possible air bubble trapping underneath each inclusion compartment was minimized by putting extra gelatin solution in the indentation before the compartment was loaded. We think this strategy was successful because images showed little evidence that bubbles were prevalent in the lower part of the phantom. In general, air bubbles tended to form on the surface of the spheres, and got exacerbated over time as mentioned in Section 4.4. We suspect that hydrophobic characteristic of the water-proof coating may be a contributor, and plan to explore other coating options to mitigate the effect in future experiments.

One limitation of our experiments was that the temperature was not controlled. Since the phantom was taken from a refrigerator shortly before each scan session, significant temperature change could have happened during the scan. Measurement under a representative experimental condition (S7 File) revealed that the temperature at the center of the phantom rose from 8.3 to 14.5°C during a typical session (including scan preparation) lasting ~90 minutes. For the purpose of comparing the present results with future experiments, we propose that the phantom temperature should be taken as 11.4 ± 3°C. There could also have been significant spatial (radial) temperature gradient; however, any center-out signal variation attributable to such gradient was not evident in the images. At high fields, the magnetic property of the iron oxide nanoparticles depends on temperature through saturation magnetization (M_s_). While references for the particular particles used (Molday ION) were not readily available, in ref [17], smaller (16 nm vs our 30 nm) iron oxide nanoparticles exhibited a temperature coefficient of M_s_ less than 1% per 10°C near the room temperature. This is more than 3 times weaker than the Curie's law prediction (for individual paramagnetic ions, ~1/T), and would imply that our experiments were subject to less than 1% error in susceptibility if the results of ref [17] are applicable. The temperature issue should be resolved if future phantoms could be stored at room temperature.

In our work, we did not attempt precise control of the inclusion sphere locations better than about a couple of millimeters, by manually scooping out on the base gel in Step 2. For better definition of the positions, one could potentially utilize a 3D-printed plastic template during the base gel formation (Step 1), which can produce controlled recess on the gel to load the small spheres.

Fig 5 shows that significant unwanted phase was introduced by environmental susceptibility effect, especially from the phantom support material touching the lower part of the phantom. To construct a support piece from lighter materials or materials with more air-like susceptibility [20, 21] will help reduce the phase contamination, and allow better exploitation of the benefits of the spherical phantom geometry.

In our phantom, formalin was added to suppress water diffusion across compartmental boundaries. In terms of magnetic susceptibility, we do not believe the effect of formalin was significant. Generally, any diamagnetic liquid with mass density similar to water has volume susceptibility close to water [1]. Since we have added 2.5 ml of formalin solution to 250 ml of gel, any susceptibility difference would have been diluted by 100, diminishing its effect. For verification, we compared the R_2_* of pure gel and gel + formalin phantoms in a separate experiment (S8 File). We found R_2_* (gel) = 2.86 s^-1^, R_2_* (gel + formalin) = 3.17 s^-1^, therefore ΔR_2_* (formalin) = 0.31 s^-1^, about 3% of the R_2_* of the lowest-density doped gel compartment (10.1 s^-1^) in the inclusion phantom. Assuming linear relationship between R_2_* and susceptibility, we estimate the formalin solution susceptibility of 3% × 0.1 ppm = 0.003 ppm, comparable to ^1^H nuclear spin susceptibility that is normally negligible [22].

In conclusion, we have demonstrated a method to fabricate a spherical gel phantom which contains smaller spherical compartments for MRI-based magnetic susceptibility imaging. The proposed method allows repeatable production of susceptibility inclusion phantoms with controlled geometry, which can be useful for experimental validation of MR phase imaging and quantitative susceptibility mapping.

## Supporting information

S1 Fig Shim-dependent phase maps. (PPTX)

S2 Fig Measured vs model phase maps for inclusion susceptibilities of 0.1, 0.119, 0.165 ppm. (PPTX)

S1 File Image (DICOM) files per sequences in Table 2. (ZIP)

S2 File Relaxation time data, 1st measurement. (PPTX)

S3 File Linear regression for iron oxide concentration-dependent relaxation rates. (XLSX)

S4 File Relaxation time data, 2nd measurement. (PPTX)

S5 File Relaxation rate change over time. (XLSX)

S6 File Linear regression for iron oxide concentration-dependent susceptibility and comparison between QSM processing codes. (XLSX)

S7 File Temperature data. (XLSX)

S8 File Gel and formalin relaxation data. (PPTX)
