# Supplementary material for: Fabrication of a spherical inclusion phantom for validation of magnetic resonance-based magnetic susceptibility imaging
Source: PLoS One. 2019 Aug 5;14(8):e0220639. doi: 10.1371/journal.pone.0220639 (PMC6681938; doi:10.1371/journal.pone.0220639)
Supplement: S1 Fig — (PPTX) [file pone.0220639.s001.pptx]

## Slide 1
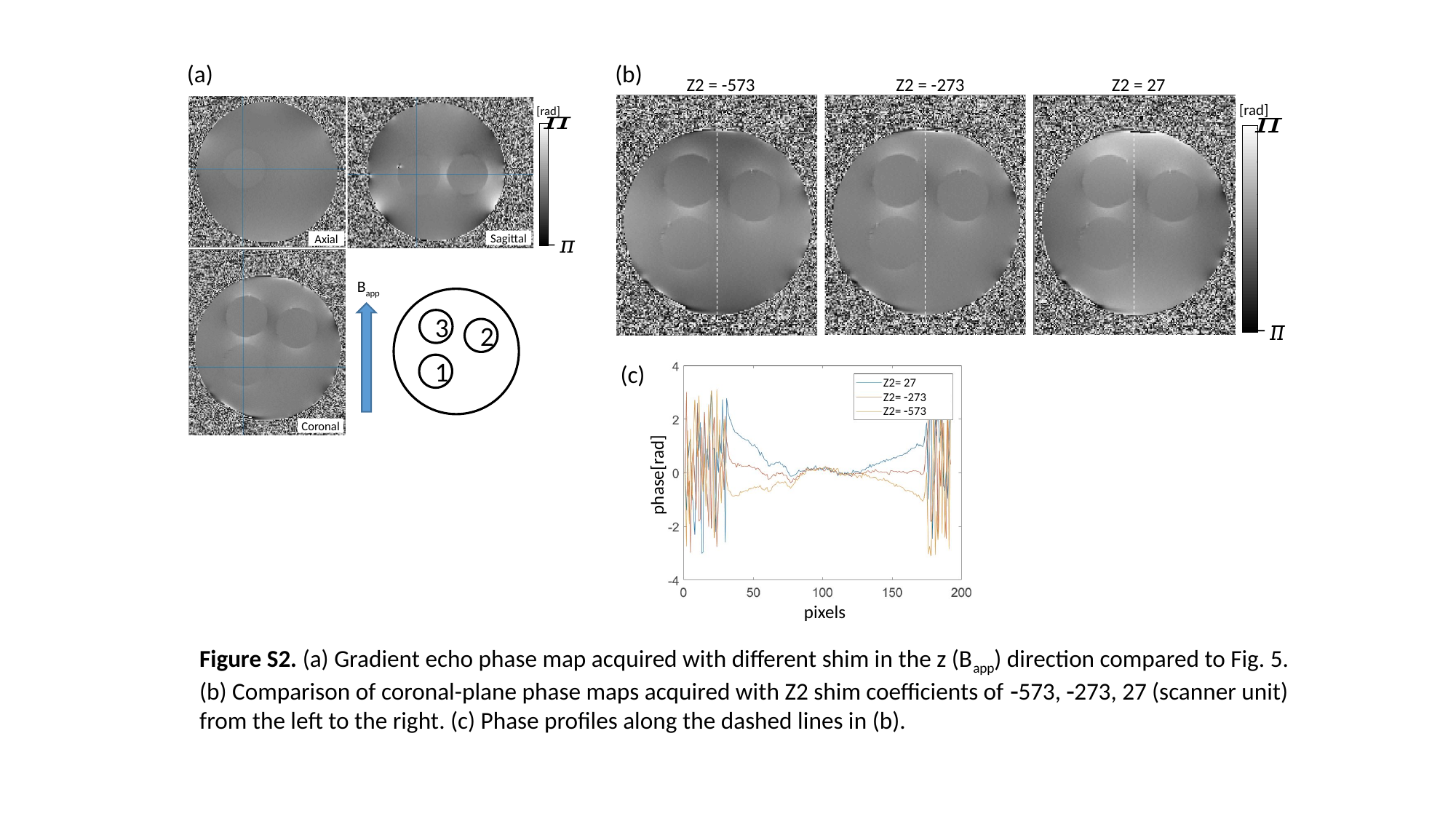

(a)
(b)
Z2 = 27
Z2 = -573
Z2 = -273
[rad]
[rad]
Sagittal
Axial
Bapp
3
2
1
Coronal
Z2= 27
Z2= −273
Z2= −573
(c)
 phase[rad]
pixels
Figure S2. (a) Gradient echo phase map acquired with different shim in the z (Bapp) direction compared to Fig. 5. (b) Comparison of coronal-plane phase maps acquired with Z2 shim coefficients of −573, −273, 27 (scanner unit) from the left to the right. (c) Phase profiles along the dashed lines in (b).
