# Supplementary material for: Fabrication of a spherical inclusion phantom for validation of magnetic resonance-based magnetic susceptibility imaging
Source: PLoS One. 2019 Aug 5;14(8):e0220639. doi: 10.1371/journal.pone.0220639 (PMC6681938; doi:10.1371/journal.pone.0220639)
Supplement: S2 Fig — (PPTX) [file pone.0220639.s002.pptx]

## Slide 1
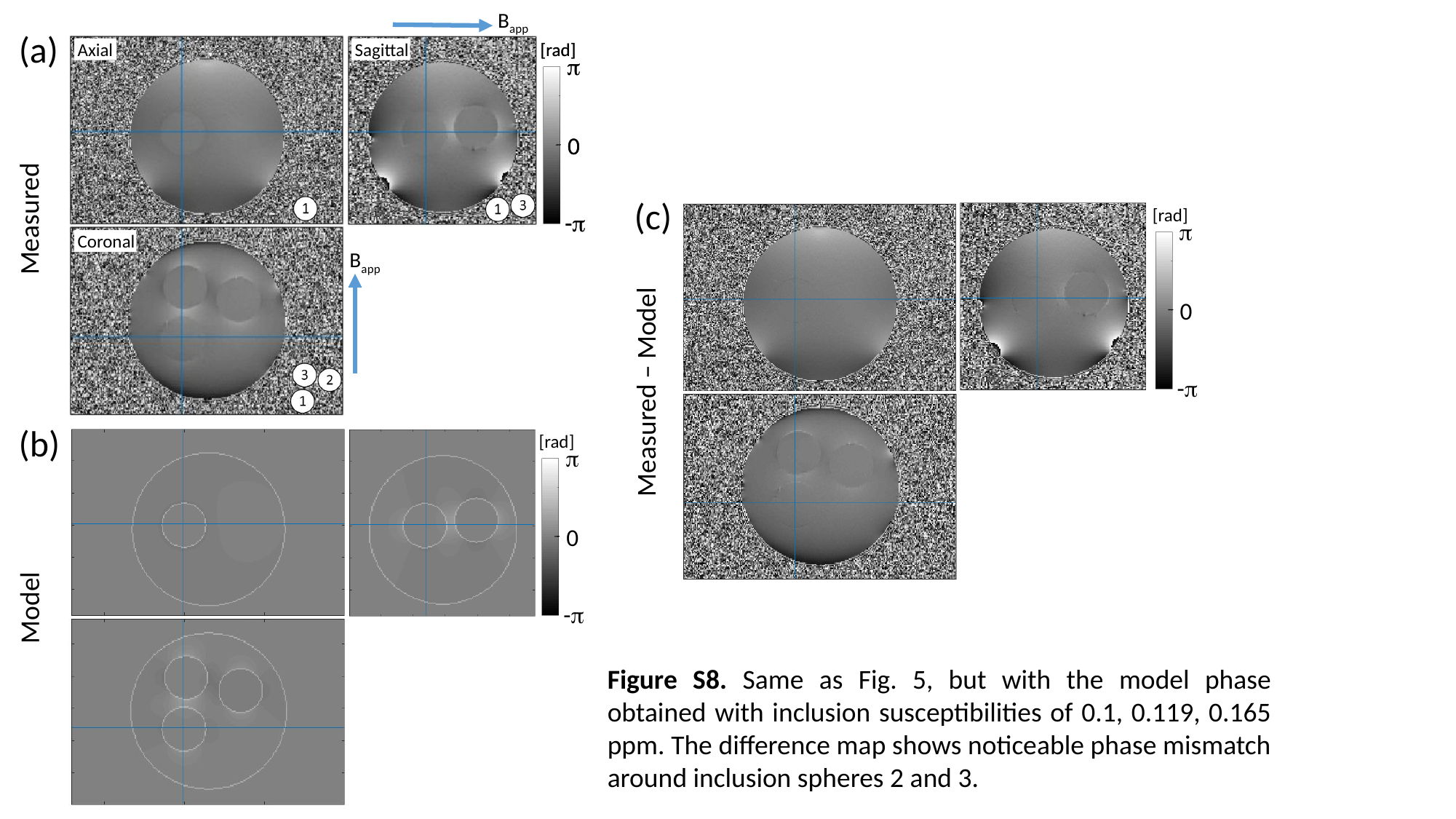

Bapp
[rad]
Axial
Sagittal

0
-
Coronal
Bapp
(a)
[rad]

0
-
Measured
(c)
[rad]

0
-
Measured ̶ Model
(b)
[rad]

0
-
Model
Figure S8. Same as Fig. 5, but with the model phase obtained with inclusion susceptibilities of 0.1, 0.119, 0.165 ppm. The difference map shows noticeable phase mismatch around inclusion spheres 2 and 3.
