# Supplementary material for: Fabrication of a spherical inclusion phantom for validation of magnetic resonance-based magnetic susceptibility imaging
Source: PLoS One. 2019 Aug 5;14(8):e0220639. doi: 10.1371/journal.pone.0220639 (PMC6681938; doi:10.1371/journal.pone.0220639)
Supplement: S1 File — (ZIP) [file pone.0220639.s003.zip › readme.docx]

1) For T1, T2, T2*, we submit all the acquired single-slice images (DICOM) for the relaxation time fitting (Total 24 images; 7, 12, 5 images for T1, T2, T2*, respectively).

2) For QSM data acquisition, due to the large number of image files (16 channels used x 8 echoes x 144 slices = 18,432 images, for magnitude and phase each), we only submit a subset, namely 16-channel magnitude images each with 2 echoes (first and 4th) on a middle axial slice (total images = 16 x 2 = 32).
