# Supplementary material for: Fabrication of a spherical inclusion phantom for validation of magnetic resonance-based magnetic susceptibility imaging
Source: PLoS One. 2019 Aug 5;14(8):e0220639. doi: 10.1371/journal.pone.0220639 (PMC6681938; doi:10.1371/journal.pone.0220639)
Supplement: S2 File — (PPTX) [file pone.0220639.s004.pptx]

## Slide 1
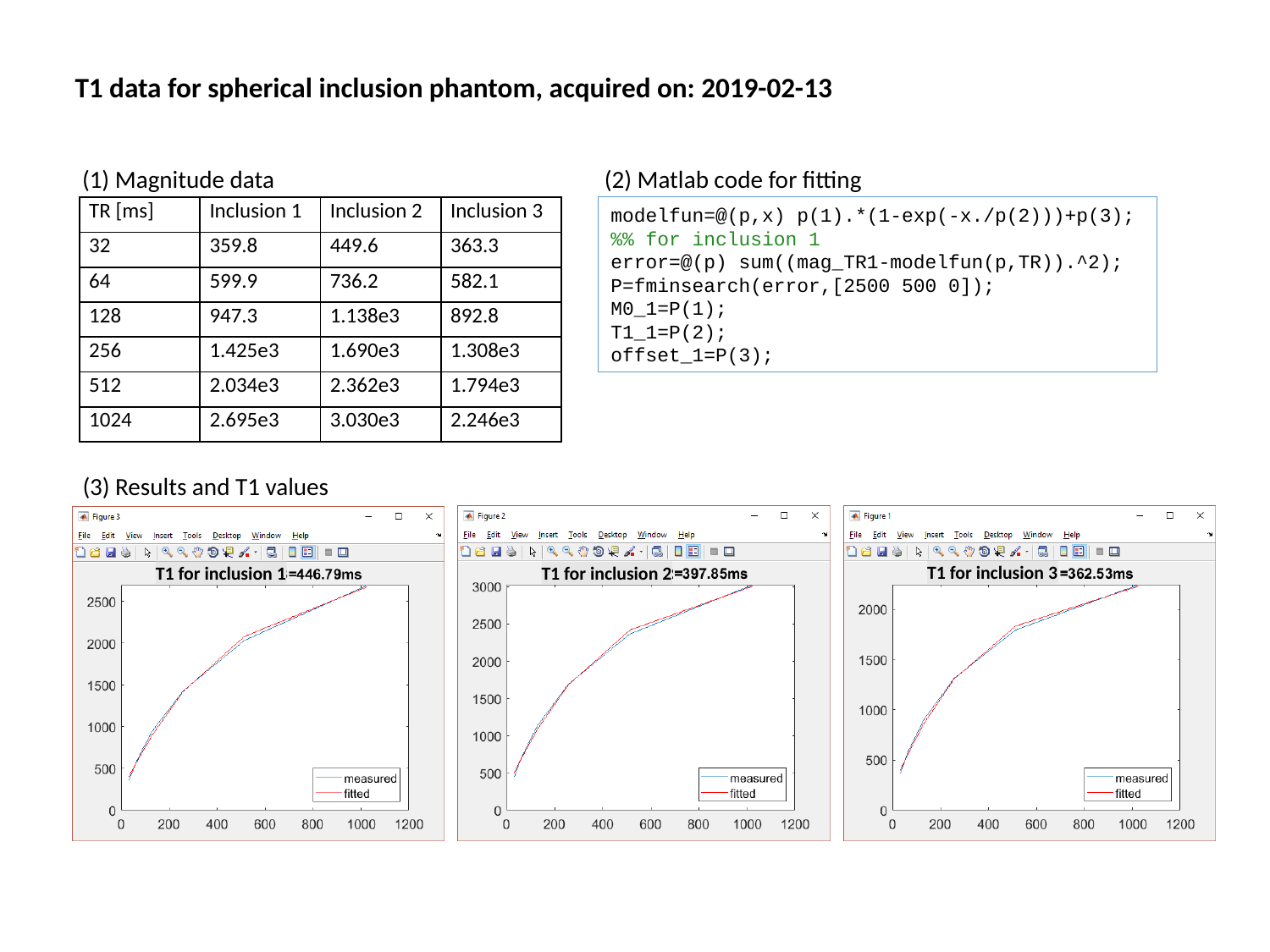

T1 data for spherical inclusion phantom, acquired on: 2019-02-13
(1) Magnitude data
(2) Matlab code for fitting
modelfun=@(p,x) p(1).*(1-exp(-x./p(2)))+p(3);
%% for inclusion 1
error=@(p) sum((mag_TR1-modelfun(p,TR)).^2);
P=fminsearch(error,[2500 500 0]);
M0_1=P(1);
T1_1=P(2);
offset_1=P(3);
| TR [ms] | Inclusion 1 | Inclusion 2 | Inclusion 3 |
| --- | --- | --- | --- |
| 32 | 359.8 | 449.6 | 363.3 |
| 64 | 599.9 | 736.2 | 582.1 |
| 128 | 947.3 | 1.138e3 | 892.8 |
| 256 | 1.425e3 | 1.690e3 | 1.308e3 |
| 512 | 2.034e3 | 2.362e3 | 1.794e3 |
| 1024 | 2.695e3 | 3.030e3 | 2.246e3 |
(3) Results and T1 values
T1 for inclusion 3
T1 for inclusion 2
T1 for inclusion 1

## Slide 2
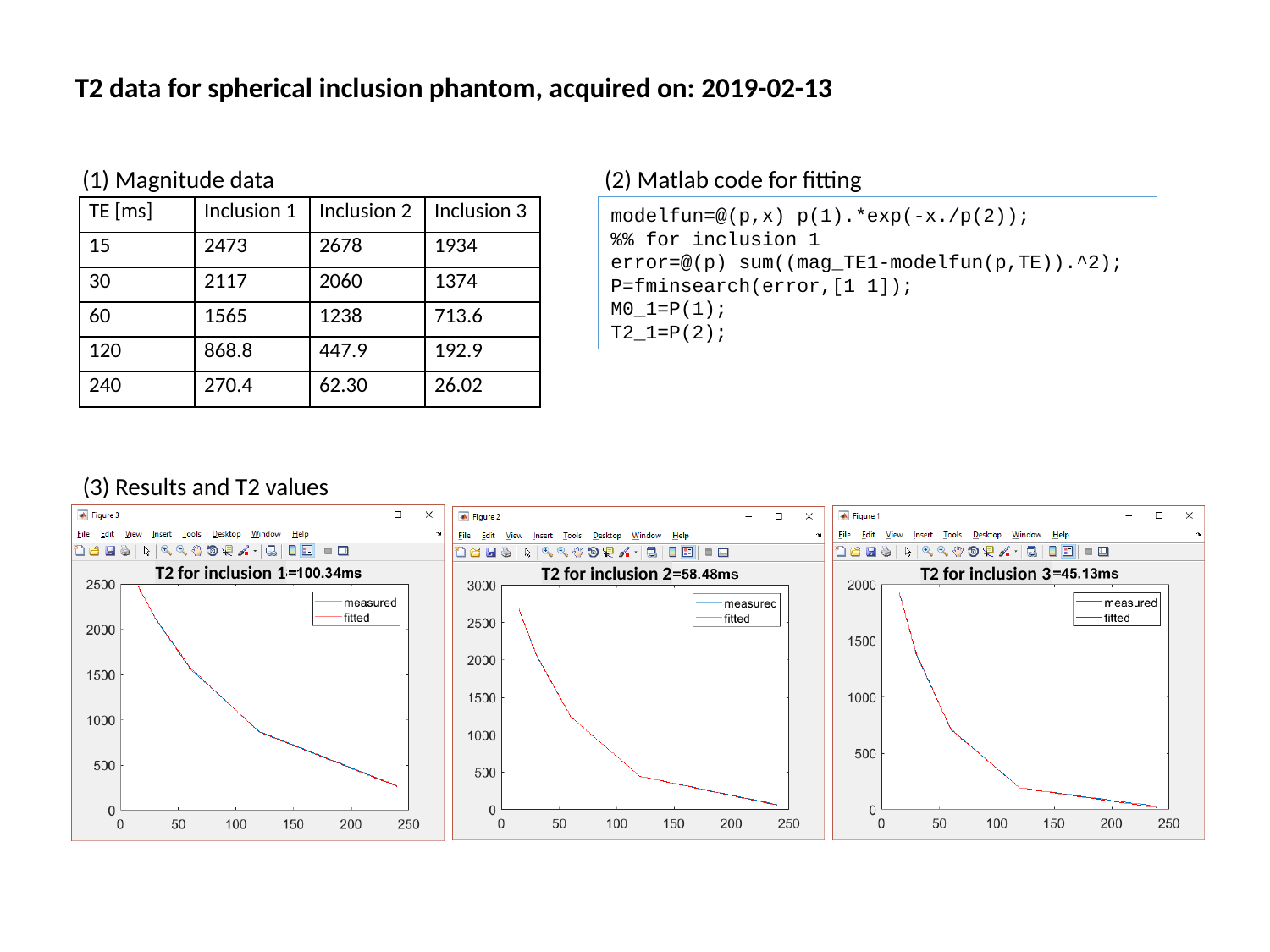

T2 data for spherical inclusion phantom, acquired on: 2019-02-13
(1) Magnitude data
(2) Matlab code for fitting
modelfun=@(p,x) p(1).*exp(-x./p(2));
%% for inclusion 1
error=@(p) sum((mag_TE1-modelfun(p,TE)).^2);
P=fminsearch(error,[1 1]);
M0_1=P(1);
T2_1=P(2);
| TE [ms] | Inclusion 1 | Inclusion 2 | Inclusion 3 |
| --- | --- | --- | --- |
| 15 | 2473 | 2678 | 1934 |
| 30 | 2117 | 2060 | 1374 |
| 60 | 1565 | 1238 | 713.6 |
| 120 | 868.8 | 447.9 | 192.9 |
| 240 | 270.4 | 62.30 | 26.02 |
(3) Results and T2 values
T2 for inclusion 1
T2 for inclusion 3
T2 for inclusion 2

## Slide 3
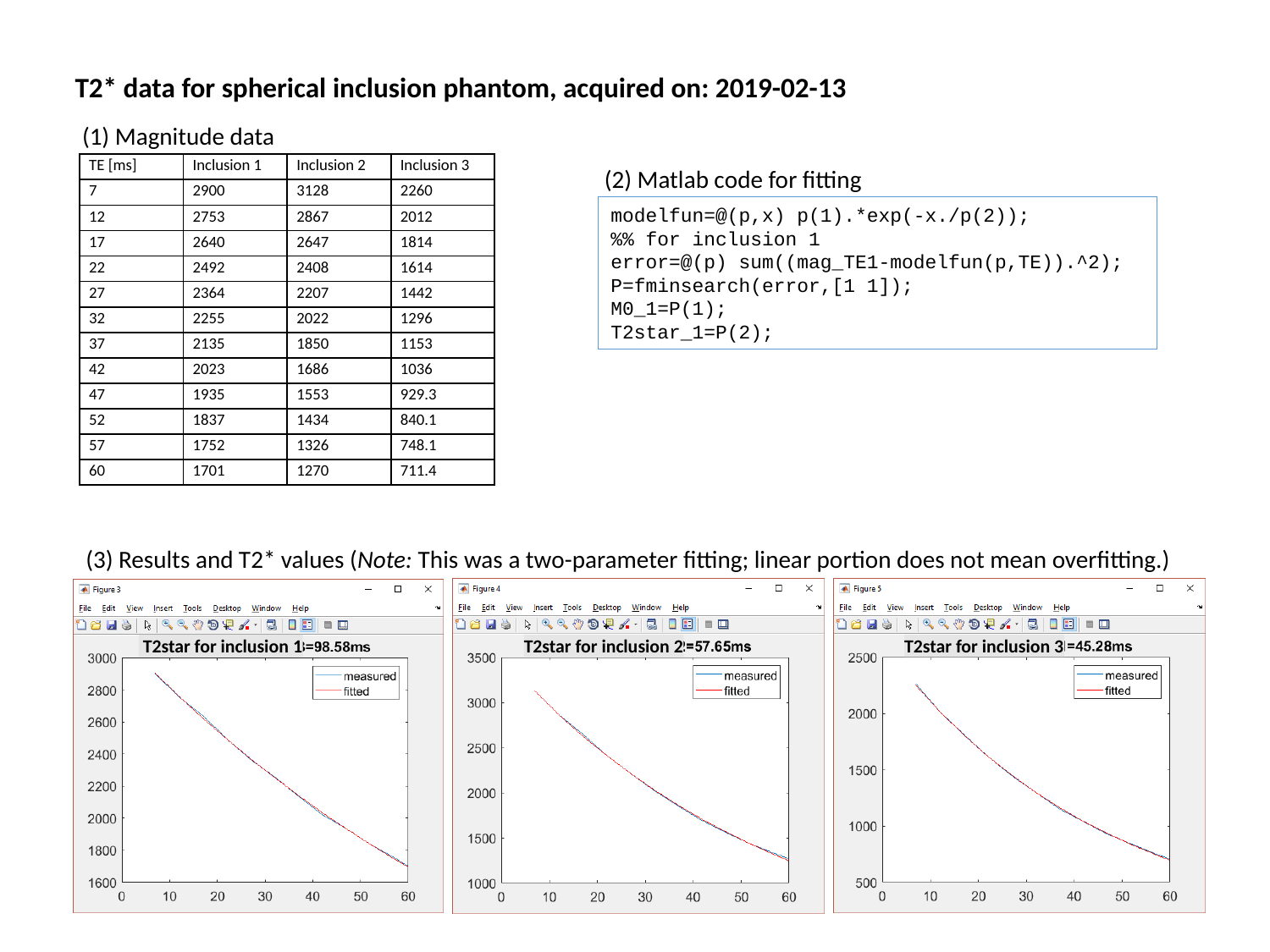

T2* data for spherical inclusion phantom, acquired on: 2019-02-13
(1) Magnitude data
| TE [ms] | Inclusion 1 | Inclusion 2 | Inclusion 3 |
| --- | --- | --- | --- |
| 7 | 2900 | 3128 | 2260 |
| 12 | 2753 | 2867 | 2012 |
| 17 | 2640 | 2647 | 1814 |
| 22 | 2492 | 2408 | 1614 |
| 27 | 2364 | 2207 | 1442 |
| 32 | 2255 | 2022 | 1296 |
| 37 | 2135 | 1850 | 1153 |
| 42 | 2023 | 1686 | 1036 |
| 47 | 1935 | 1553 | 929.3 |
| 52 | 1837 | 1434 | 840.1 |
| 57 | 1752 | 1326 | 748.1 |
| 60 | 1701 | 1270 | 711.4 |
(2) Matlab code for fitting
modelfun=@(p,x) p(1).*exp(-x./p(2));
%% for inclusion 1
error=@(p) sum((mag_TE1-modelfun(p,TE)).^2);
P=fminsearch(error,[1 1]);
M0_1=P(1);
T2star_1=P(2);
(3) Results and T2* values (Note: This was a two-parameter fitting; linear portion does not mean overfitting.)
T2star for inclusion 3
T2star for inclusion 2
 T2star for inclusion 1
