# Supplementary material for: Fabrication of a spherical inclusion phantom for validation of magnetic resonance-based magnetic susceptibility imaging
Source: PLoS One. 2019 Aug 5;14(8):e0220639. doi: 10.1371/journal.pone.0220639 (PMC6681938; doi:10.1371/journal.pone.0220639)
Supplement: S4 File — (PPTX) [file pone.0220639.s006.pptx]

## Slide 1
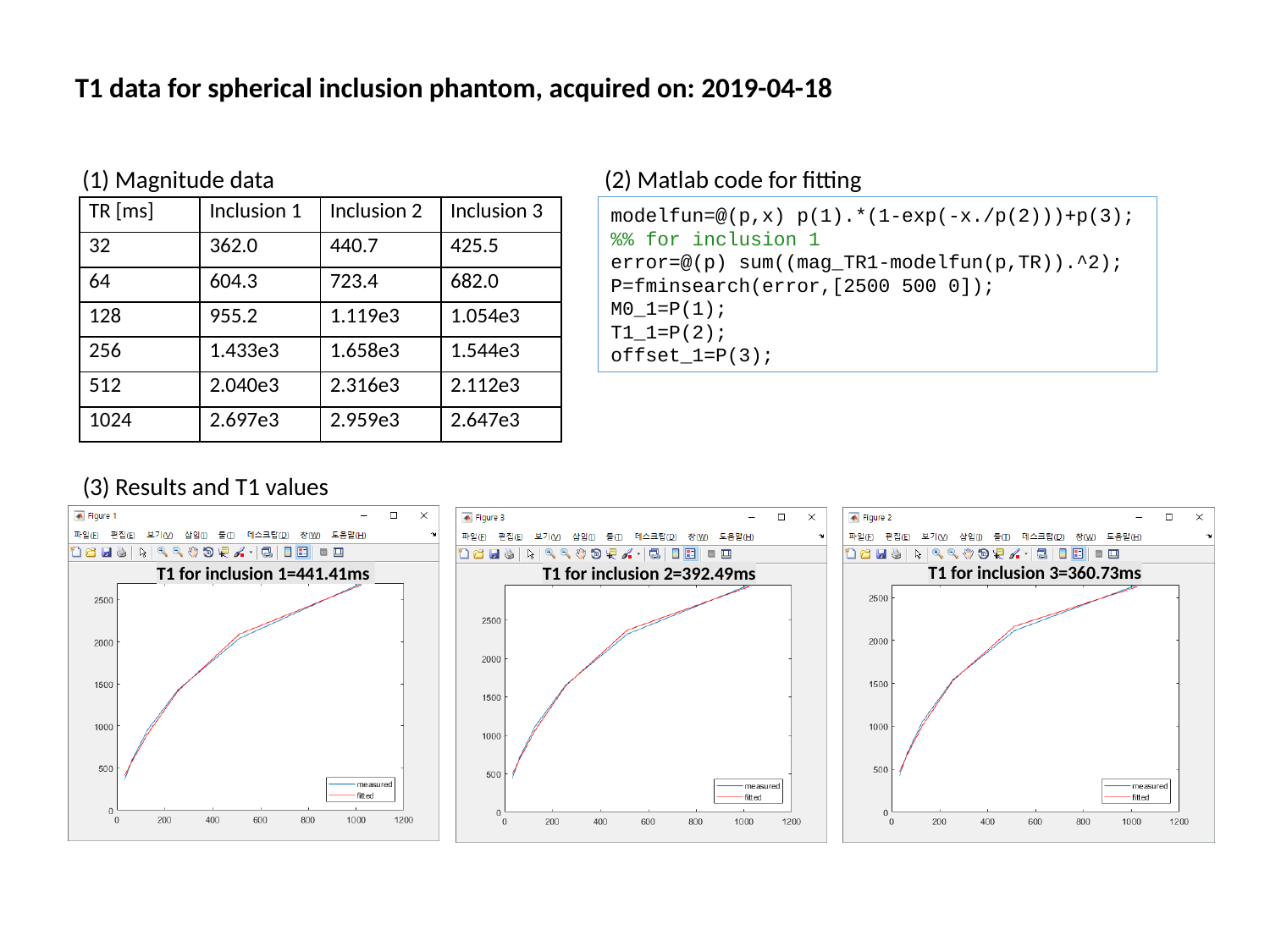

T1 data for spherical inclusion phantom, acquired on: 2019-04-18
(1) Magnitude data
(2) Matlab code for fitting
modelfun=@(p,x) p(1).*(1-exp(-x./p(2)))+p(3);
%% for inclusion 1
error=@(p) sum((mag_TR1-modelfun(p,TR)).^2);
P=fminsearch(error,[2500 500 0]);
M0_1=P(1);
T1_1=P(2);
offset_1=P(3);
| TR [ms] | Inclusion 1 | Inclusion 2 | Inclusion 3 |
| --- | --- | --- | --- |
| 32 | 362.0 | 440.7 | 425.5 |
| 64 | 604.3 | 723.4 | 682.0 |
| 128 | 955.2 | 1.119e3 | 1.054e3 |
| 256 | 1.433e3 | 1.658e3 | 1.544e3 |
| 512 | 2.040e3 | 2.316e3 | 2.112e3 |
| 1024 | 2.697e3 | 2.959e3 | 2.647e3 |
(3) Results and T1 values
T1 for inclusion 3=360.73ms
T1 for inclusion 2=392.49ms
T1 for inclusion 1=441.41ms

## Slide 2
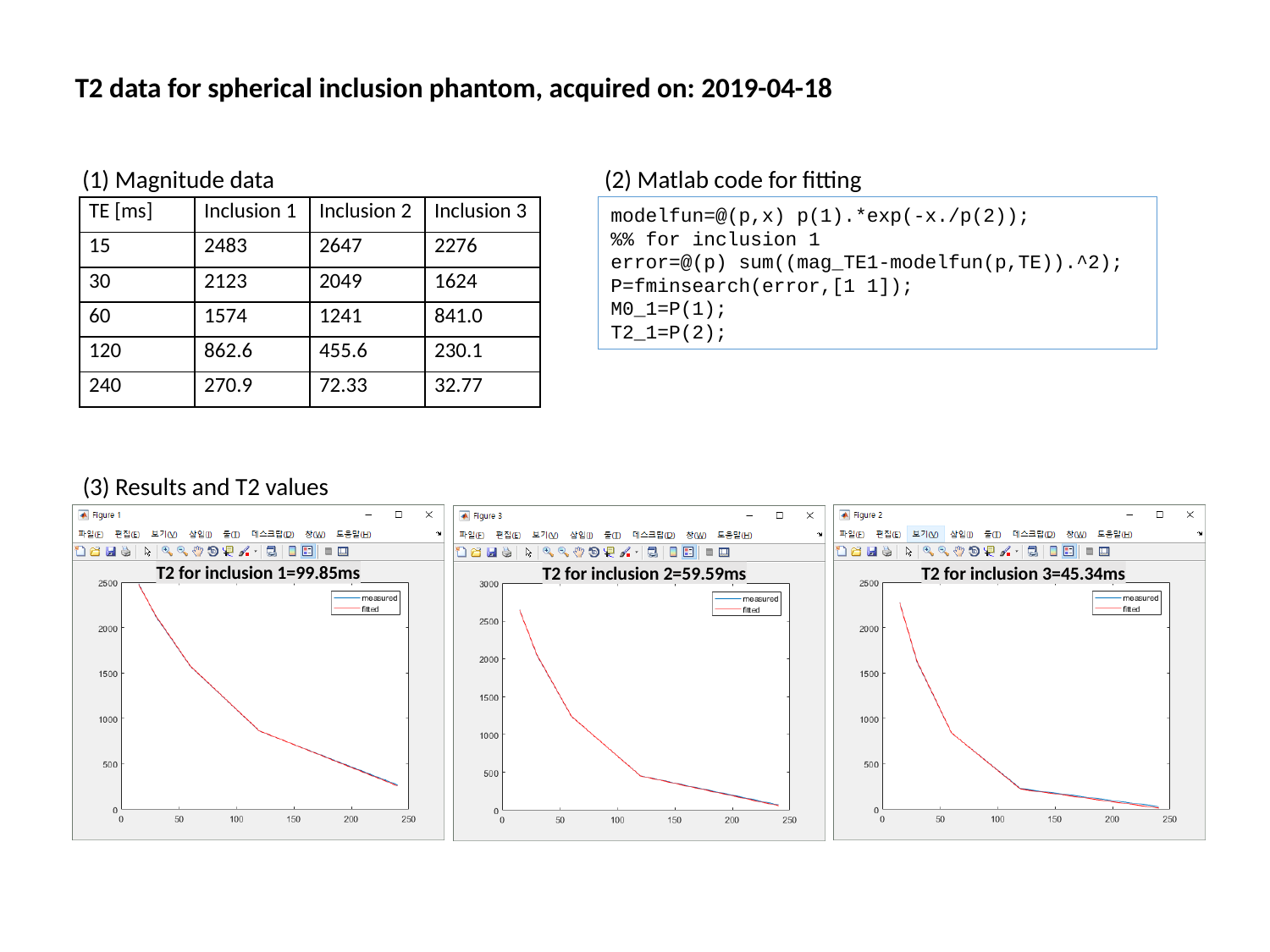

T2 data for spherical inclusion phantom, acquired on: 2019-04-18
(1) Magnitude data
(2) Matlab code for fitting
modelfun=@(p,x) p(1).*exp(-x./p(2));
%% for inclusion 1
error=@(p) sum((mag_TE1-modelfun(p,TE)).^2);
P=fminsearch(error,[1 1]);
M0_1=P(1);
T2_1=P(2);
| TE [ms] | Inclusion 1 | Inclusion 2 | Inclusion 3 |
| --- | --- | --- | --- |
| 15 | 2483 | 2647 | 2276 |
| 30 | 2123 | 2049 | 1624 |
| 60 | 1574 | 1241 | 841.0 |
| 120 | 862.6 | 455.6 | 230.1 |
| 240 | 270.9 | 72.33 | 32.77 |
(3) Results and T2 values
T2 for inclusion 1=99.85ms
T2 for inclusion 2=59.59ms
T2 for inclusion 3=45.34ms

## Slide 3
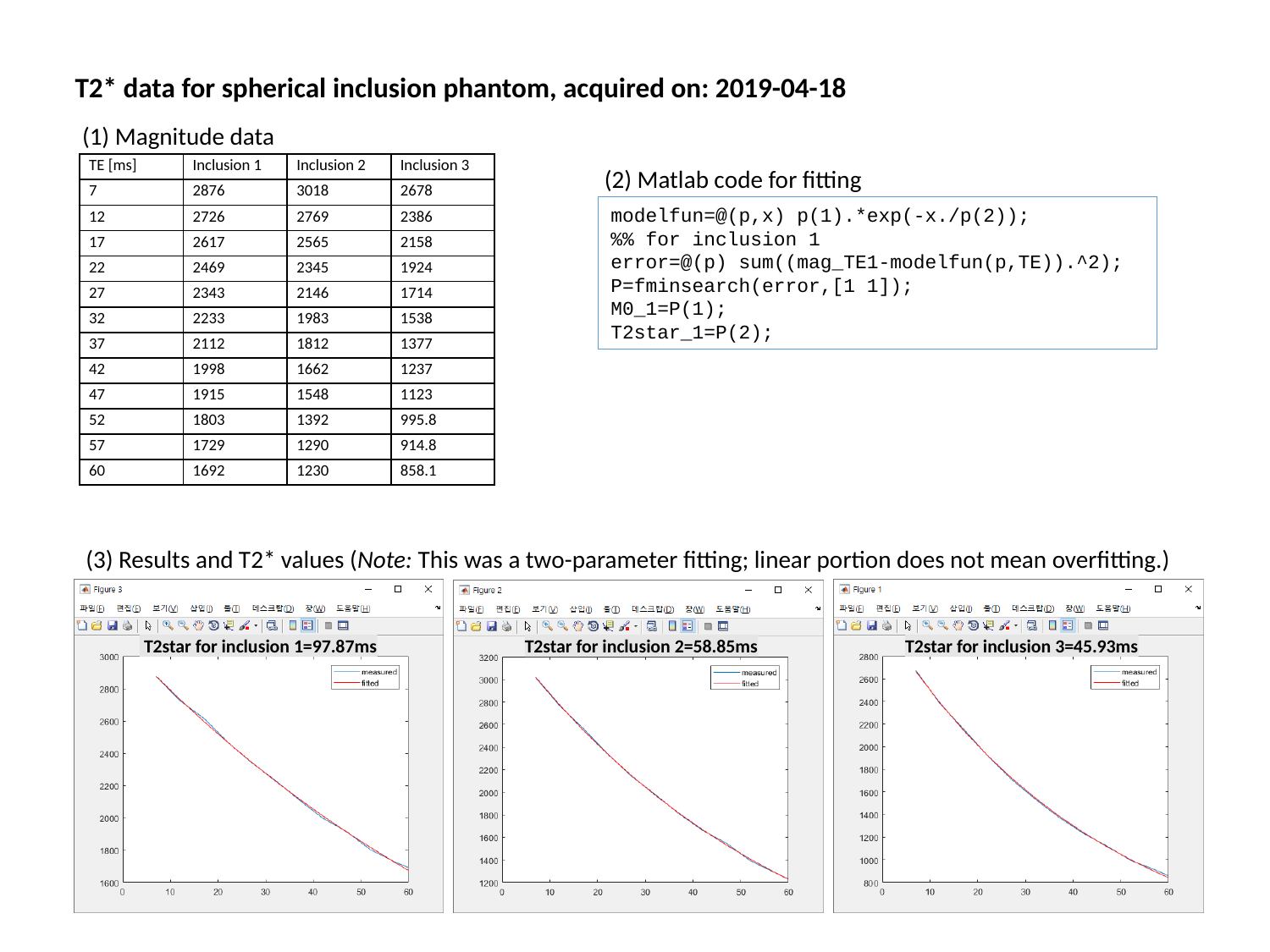

T2* data for spherical inclusion phantom, acquired on: 2019-04-18
(1) Magnitude data
| TE [ms] | Inclusion 1 | Inclusion 2 | Inclusion 3 |
| --- | --- | --- | --- |
| 7 | 2876 | 3018 | 2678 |
| 12 | 2726 | 2769 | 2386 |
| 17 | 2617 | 2565 | 2158 |
| 22 | 2469 | 2345 | 1924 |
| 27 | 2343 | 2146 | 1714 |
| 32 | 2233 | 1983 | 1538 |
| 37 | 2112 | 1812 | 1377 |
| 42 | 1998 | 1662 | 1237 |
| 47 | 1915 | 1548 | 1123 |
| 52 | 1803 | 1392 | 995.8 |
| 57 | 1729 | 1290 | 914.8 |
| 60 | 1692 | 1230 | 858.1 |
(2) Matlab code for fitting
modelfun=@(p,x) p(1).*exp(-x./p(2));
%% for inclusion 1
error=@(p) sum((mag_TE1-modelfun(p,TE)).^2);
P=fminsearch(error,[1 1]);
M0_1=P(1);
T2star_1=P(2);
(3) Results and T2* values (Note: This was a two-parameter fitting; linear portion does not mean overfitting.)
T2star for inclusion 3=45.93ms
T2star for inclusion 2=58.85ms
 T2star for inclusion 1=97.87ms
