# Supplementary material for: Fabrication of a spherical inclusion phantom for validation of magnetic resonance-based magnetic susceptibility imaging
Source: PLoS One. 2019 Aug 5;14(8):e0220639. doi: 10.1371/journal.pone.0220639 (PMC6681938; doi:10.1371/journal.pone.0220639)
Supplement: S8 File — (PPTX) [file pone.0220639.s010.pptx]

## Slide 1
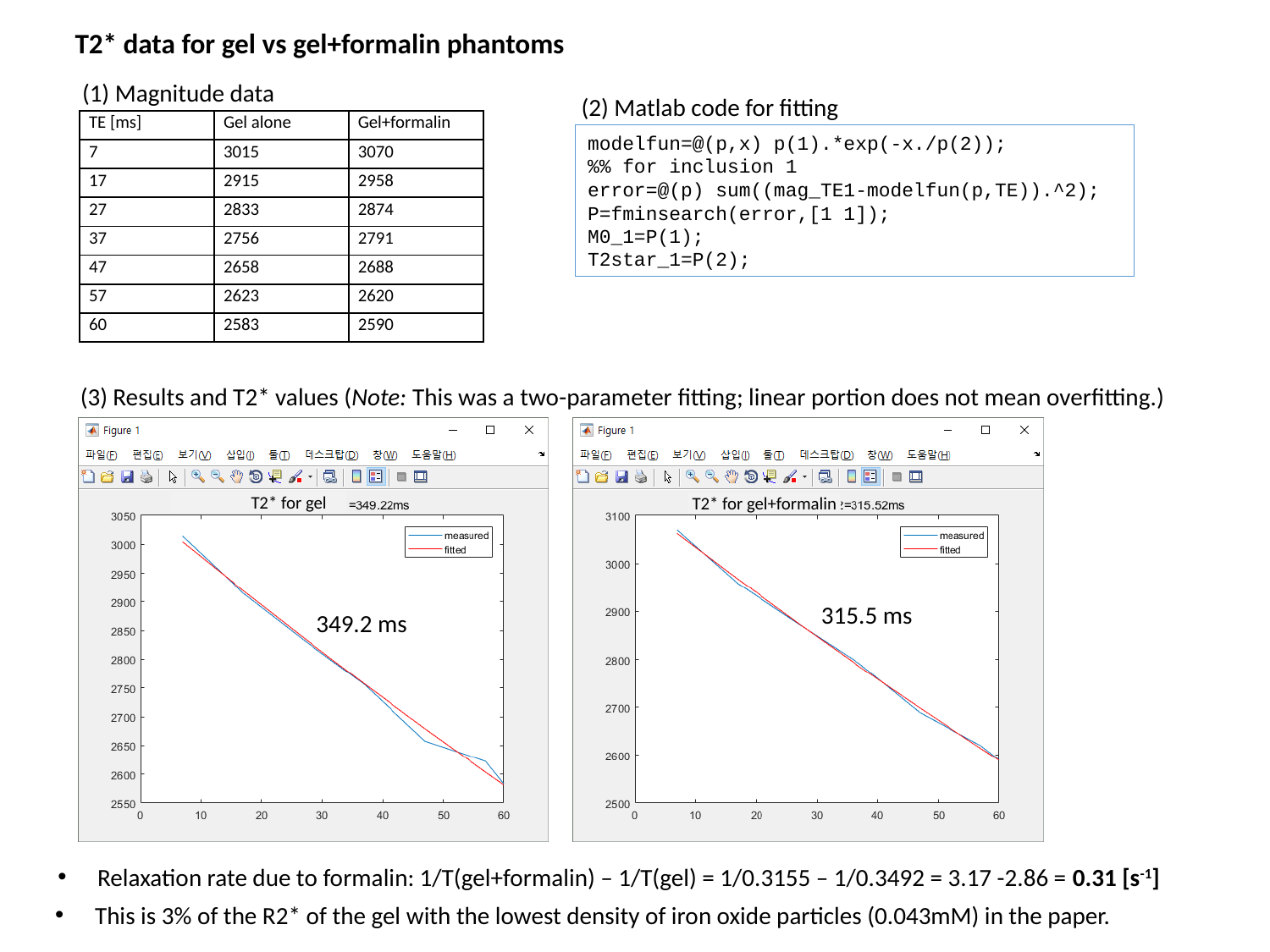

T2* data for gel vs gel+formalin phantoms
(1) Magnitude data
(2) Matlab code for fitting
| TE [ms] | Gel alone | Gel+formalin |
| --- | --- | --- |
| 7 | 3015 | 3070 |
| 17 | 2915 | 2958 |
| 27 | 2833 | 2874 |
| 37 | 2756 | 2791 |
| 47 | 2658 | 2688 |
| 57 | 2623 | 2620 |
| 60 | 2583 | 2590 |
modelfun=@(p,x) p(1).*exp(-x./p(2));
%% for inclusion 1
error=@(p) sum((mag_TE1-modelfun(p,TE)).^2);
P=fminsearch(error,[1 1]);
M0_1=P(1);
T2star_1=P(2);
(3) Results and T2* values (Note: This was a two-parameter fitting; linear portion does not mean overfitting.)
 T2* for gel+formalin
315.5 ms
 T2* for gel
349.2 ms
Relaxation rate due to formalin: 1/T(gel+formalin) – 1/T(gel) = 1/0.3155 – 1/0.3492 = 3.17 -2.86 = 0.31 [s-1]
This is 3% of the R2* of the gel with the lowest density of iron oxide particles (0.043mM) in the paper.
